# Orphan Cytochromes P450 as Possible Pharmacological Targets or Biomarkers in Breast Cancer

**DOI:** 10.3390/cimb47090682

**Published:** 2025-08-25

**Authors:** Barbara Licznerska, Hanna Szaefer, Wanda Baer-Dubowska

**Affiliations:** Department of Pharmaceutical Biochemistry, Poznan University of Medical Sciences, 60–806 Poznań, Poland; barlicz@ump.edu.pl (B.L.); baerw@ump.edu.pl (W.B.-D.)

**Keywords:** orphan CYPs 450, CYP4Z1, CYP2S1, CYP2W1, CYP2U1, CYP4X1, breast cancer treatment

## Abstract

Although significant advances in the treatment of breast cancer have been made over the last few decades, searching for more effective prophylaxis and therapy for this type of cancer is still topical. Orphan cytochromes (CYPs) P450 are enzymes whose functions and substrates are not fully known. The overexpression of some orphan CYPs in breast cancer tissue warrants attention as a possible breast cancer prophylaxis/treatment target or biomarker. Of particular interest is CYP4Z1, which seems to be specific for breast cancer, including triple-negative breast cancer (TNBC). The currently available data indicate that inhibition of CYP4Z1 breast-specific expression may reduce the growth, progression, angiogenesis, and invasiveness of breast cancer. Although less specific, the other orphan CYPs, such as CYP2W1, CYP2S1, CYP2U1, and CYP4X1, exhibit significantly higher expression in breast tumors compared to normal tissues. The available data indicate that these CYP isoforms catalyze the hydroxylation of fatty acids. Their products, such as epoxyeicosatrienoic acids (EETs) or hydroxyeicosatetraenoic acids (HETEs), are considered critical modulators of cancer progression. Therefore, inhibition of the expression and activity of these orphan CYPs might be more useful in cancer treatment than in prophylaxis. This review summarizes current knowledge of orphan CYPs in breast tissue and their possible application in drug targeting or prognosis assessment.

## 1. Introduction

Cytochromes P450 (CYPs) catalyze a variety of reactions and are of significant importance in the metabolism of xenobiotics, including drugs and carcinogens. It is estimated that ~75% of drug metabolism reactions involve CYPs, and ~66% of carcinogens are bioactivated by this class of enzymes [1]. CYP enzymes play a crucial role in the initiation of carcinogenesis through the activation of various environmental and endogenous carcinogens, including estrogen metabolites, as well as in the later stages of cancer development. Therefore, CYPs are considered promising targets in cancer prophylaxis and chemotherapy. Moreover, they may activate or inactivate prodrugs [2,3]. While most of these reactions involve CYPs with a well-characterized function and substrate specificity, it is now clear that some orphan CYPs may be equally important for the transformation of drugs and carcinogens. The term ‘orphan CYPs’ was adopted from descriptions of the steroid nuclear superfamily [4], and it is estimated that out of the 57 human cytochromes P450 (P450) and 58 pseudogenes discovered to date, 1/4 remain ’orphans‘ in the sense that their function, expression sites, and regulation are still largely not elucidated [5]. Interestingly, the expression of some of these orphan CYPs is tissue-specific and increases in the process of tumorigenesis. For example, CYP4Z1 was found to be frequently upregulated in primary mammary carcinoma and ovarian cancer, and it was associated with tumor progression and metastasis [6,7]. The others, such as CYP2W1, CYP2S1, CYP2U1, CYP4X1, and CYP4V2, are less specific, but their overexpression in breast cancer has also been found. Attempts are being made to deorphanize some of them, but so far, these are only partly successful [8].

Breast cancer is the leading cause of cancer-related deaths among women worldwide. Although breast cancer mortality has been dropping since the 1990s [9], there is still much to be done to reduce these numbers. Estrogens play a key role in the pathogenesis of breast cancer. Immunohistochemically, breast cancer has been classified into four subtypes: estrogen receptor-positive (ER+), progesterone receptor-positive (PR+), human epidermal growth factor receptor 2-positive (HER+), and triple-negative (TNBC) [10]. TNBC accounts for 10–20% of all cases and is resistant to conventional endocrine therapy [11]. Therefore, there is a particularly unmet need for highly effective therapeutic agents against TNBC. Thus, new druggable targets, such as the orphan CYPs expressed in breast epithelial cells, are of particular interest in targeted breast cancer prophylaxis and therapy [12]. This review focuses on the description and discussion of current orphan CYP knowledge in breast tissue and the prospects for further investigation into their deorphanization and possible application as targets or biomarkers for the prevention and/or treatment of breast cancer. Based on the available data, orphan CYPs, the most promising in this context, were selected.

## 2. Expression of Orphan CYPs in Breast Epithelium and Cancer

Most data on the expression of orphan CYPs’ genes or enzyme activity in the breast epithelium come from in vitro studies using breast cancer cell lines derived from cancers differing in tumor origin and receptor status. In the most extensively investigated ER(+) MCF7 breast cancer cell line, the expression of three orphan CYPs, namely CYP4Z1, CYP2S1, and CYP2W1, was confirmed by several authors [13,14,15,16,17,18,19,20]. The analysis of the CYP expression profile in the ER(+) T47D cell line, also derived from ductal carcinoma, revealed the presence of CYP4Z1 [7,16,21,22]. A stable expression of CYP4Z1 was achieved in BT-474 human HER(+) breast cancer cells [21]. The CYP4Z1 isoform has triggered particular interest because of its hypothetical role in breast cancer through the formation of the signaling molecule 20-hydroxyeicosatetraenoic acid (20-HETE). The expression of this orphan isoform, as well as of CYP2S1 and CYP2W1, was also described in triple-negative cell lines MDA-MB-231 and MDA-MB-468 [13,14,17,18]. Moreover, in comparison to ER(+) and PR(+) breast cancer cells, higher levels of orphan CYPs’ expression were observed in these cell lines. Concurrently, in the nontumorigenic breast epithelial MCF10A cell line, several orphan CYPs’ mRNA were detected, in particular CYP2S1, CYP2U1, CYP4V2, CYP4X1, and CYP4Z1. However, the CYP4Z1 protein was not revealed on the MCF10A cells’ surface [23,24].

To establish the possible role of orphan CYPs in breast cancer development and/or chemotherapy resistance, Tao Xi et al. [17,25,26] analyzed the synergic expression of CYP4Z1 and pseudogene CYP4Z2P in the regular MCF7 cells, tamoxifen-resistant MCF7-TamR, and MDA-MB-231 cell lines. They concluded that overexpression of CYP4Z1 and/or 4Z2P might enhance the transcriptional ERα activity, apoptosis, stemness, and resistance to tamoxifen of breast cancer cells. These observations confirmed a study that used a human transgenic model, and showed that overexpression of CYP4Z1 in lactating female transgenic mice did not result in tumor formation or other mammary abnormalities, but upregulated estrogen receptor (ERα) expression was observed [27]. Additionally, in vitro studies of this group suggested that human CYP4Z1 might metabolize a small molecule into a transcriptional activator of ERα. Moreover, stable overexpression of CYP4Z1 in breast cancer cells has been reported to promote angiogenesis and tumor growth in mice [21]. Table 1 summarizes the in vitro studies on the orphan CYPs in human breast epithelial cell lines.

A more recent clinical study showed a high incidence of CYP4Z1 expression in TNBC patients with advanced grades, later stages, and larger tumors [31]. Clinical studies of the expression profiles of the other orphan CYPs and their relationship with clinical–pathological variables, although limited, showed an interesting trend. Murray et al. [6] performed immunostaining of a tissue microarray containing 170 breast cancers of no special type for a panel of 21 CYPs. The highest percentage of strong immunopositivity in these samples was seen for CYP4X1, CYP2S1, and CYP2U1. At the same time, CYP4V2, CYP4X1, and CYP4Z1 showed correlation with the tumor grade. An association with survival was identified for CYP2S1, CYP3A4, CYP4V2, and CYP26A1; however, none of these P450s was an independent prognosis biomarker [6]. In some smaller pools of patients, increased transcript levels of CYP2S1, CYP2W1, and CYP4F11 were found in breast cancer, adjacent, and normal breast cells. Unfortunately, protein levels were inappropriately low to confirm the results [32,33]. Table 2 presents the results of the orphan CYP analyses in clinical samples.

As mentioned above, considerable attention is focused on the role of CYP4Z1 and its associated pseudogene, CYP4Z2P, due to their breast-epithelium-specific expression. In this regard, Cizkova et al. [43] noticed, in a series of 249 ER(+) breast cancer patients, a correlation between the mutation status of the *PIK3CA* oncogene and overexpression of these CYPs.

Finally, the expression of CYP2A7, an orphan CYP that raises far less interest in the context of breast cancer, showed an association with poorer survival of TNBC patients [35].

## 3. Orphan CYPs as Therapeutic Targets or Biomarkers—Mechanistic Studies

As was described in the previous section, several orphan CYPs were detected in the breast epithelium. Here, the most studied isoforms’ characteristics and their potential for prevention or therapeutic purposes in breast cancer are provided.

### 3.1. CYP4Z1

Some experimental data, including an analysis of the Human Protein Atlas for CYP4Z1 expression in normal and cancer tissues, confirm that CYP4Z1 is mainly localized in the breast and that its activity is significantly increased in breast cancer [7]. Therefore, modulating CYP4Z1 activity appears to be a promising target, even a potential ‘silver bullet’, for breast cancer treatment [46]. The CYP4Z1 gene is situated on chromosome 1p33 within a cluster containing the CYP4A11 and CYP4X1 genes. CYP4Z1 shares 54% and 52% sequence identity with CYP4X1 and CYP4A11, respectively [16]. Comparative genomic analysis shows that tumor-specific expressed sequences like CYP4Z1 are either evolutionarily new (primates or humans) or relatively young (mammals) [47]. As a result, no orthologs have been found in mice or rats, indicating that CYP4Z1 is likely specific to humans and primates [48], which somewhat limits the use of animal models. However, this also enables the development of human transgenic models, as mentioned earlier. Designing suitable small molecules as potential drugs or chemopreventive agents requires understanding of the metabolic role of CYP4Z1 in breast cancer progression and identification of its substrates. Notably, unlike other members of the CYP4 family, CYP4Z1 features heme that is not covalently linked [49].

The first functional study of CYP4Z1 was performed in 2009 by Bureik’s research group. Using human CYP4Z1 expressed in yeast, they found a unique pattern of metabolites with lauric acid generating mainly the 8-hydroxy (ω-4) product and myristic acid forming 12-hydroxy (ω-2) [50]. Subsequent studies focused on the possibility of generation by CYP4Z1 of arachidonic acid metabolites such as pro-angiogenic epoxyeicosatrienoic acids (EETs) and HETE acids, considered critical modulators of cancer progression, acting in concert with the endothelial growth factor and other growth factors, ultimately promoting cellular proliferation, neovascularization, angiogenesis, and metastasis. Of particular interest is 20-HETE, which, as was postulated, is responsible for proliferative, angiogenic, and tumor growth effects [21].

As CYP4Z1 is considered an EET synthase, it was therefore not surprising that the major products of internal oxidation of arachidonic acid (AA) in breast ductal carcinoma T47D cells engineered to express CYP4Z1 were derivatives 14,15-EET and 14,15-dihydroxy eicosatrienoic acid. Only a trace of 20-HETE was detected, leading to the conclusion that CYP4Z1 is distinct from other CYP4 enzymes and 20-HETE is not the major AA metabolite produced in a reaction catalyzed by CYP4Z1 [7,22].

Moreover, a fatty acid screening for the other substrates was performed in yeast enzyme bags (permeabilized cells from recombinant fission yeast cells), and it indicated the ability of CYP4Z1 to catalyze 11-*O*-dealkylation cleavages and 2-hydroxylation reactions of pro-luciferin compounds [7,29,51]. Through docking experiments and site-directed mutagenesis, Asn381 and Arg487 were pointed out as key active sites of CYP4Z1 [7].

The subsequent search for inhibitors was based on the fatty acid hydroxylase activity of CYP4Z1. One of the tested compounds was N-hydroxy-N′-(-butyl-2-methylphenyl)-formamidine (HET0016), a highly potent inhibitor of 20-HETE synthase. Although the results of initial studies [21] were promising, further investigations indicated only weak inhibition of CYP4Z1 [52]. This is in contrast to the results obtained with the other members of the CYP4 family and might be related to their covalent linkage with heme. The first selective mechanism-based CYP4Z1 inhibitor was discovered by Kowalski et al. in 2020, namely 8-[(1H-benzotriazol-1-y)amino]octanoic acid [22]. An analysis of major metabolites of this compound in rat plasma showed that products of its β-oxidation have a higher inhibitory effect than the parent compound [7]. One of the latest proposed CYP4Z1 inhibitors is a derivative of HET0016 with an esterified 4-carbon carboxylate tail [53]. This compound, tested in MCF7 and MDA-MB-231 cells, showed the ability to reduce metastatic potential, spheroid formation, and expression of stemness markers. The low constitutive expression of CYP4Z1 in these cell lines requires careful interpretation of these data, although even minimal activity of this enzyme might be sufficient to obtain the desired effect [7]. Previously, Rieger et al. [15] suggested utilization of the tissue- and cancer-specific expression of CYP4Z1 to bioactivate prodrugs into active agents for breast carcinoma treatment. However, despite attempts, no fruitful results have been obtained so far that would allow the use of CYP4Z1 activity to activate anti-breast-cancer prodrugs.

The observation that MCF7 breast cancer cells, in contrast to nontumorigenic MCF10A cells, display CYP4Z1 on their surface might indicate its usefulness in breast cancer immunotherapy [24].

### 3.2. CYP2S1

As was mentioned in Section 2, orphan CYP2S1 was reported to be upregulated in breast cancer cells and clinical samples. Moreover, its association with patient survival was found [36]. However, CYP2S1, in contrast to CYP4Z1, is not unequivocally linked with this tissue. Therefore, the data on its usefulness as a drug in breast cancer was not extensively studied.

The human CYP2S1 gene is located on chromosome 19, in the 19q13.2 region, encodes a protein of 504 amino acids [54], and is mainly expressed in the endoplasmic reticulum. The highest expression of CYP2S1 was found in the epithelium of portal entry organs, but a lower expression was described in several organs, including the breast epithelium. CYP2S1 catalyzes the oxidation of AA into 19-hydroxy-5Z, 8Z, 11Z, and 14Z-eicosatetraenoic acid, among other polyunsaturated fatty acids (PUFAs) with ω-1-PUFA [55]. CYP2S1 also shows epoxygenase activity involved in the metabolism of prostaglandins, modulating the inflammatory process [56].

Similarly as CYP1 family member, the induction of CYP2S1 is regulated by aryl hydrocarbon receptor (AhR) and AhR nuclear translocator (ARNT) [57]. Moreover, CYP2S1 peroxidase activity linked this CYP isoform with the metabolism of xenobiotics, such as environmental toxins/pollution and small-molecule drugs [58]. CYP2S1 can activate some anticancer prodrugs, e.g., ellipticine to 12-hydroxyellipticine and 13-hydroxyellipticine [8,59,60,61]. Therefore, it might be considered a more general therapeutic target. An analysis of the antitumor activity of GW-610 (2-(3,4-dimethoxyphenyl)-5-fluorobenzothiazole) and 5F-203 (2-(4-amino-3-methylphenyl)-5-fluorobenzothiazole) in breast cancer cell lines selectively depleted of CYP1A1, CYP2S1, and CYP2W1 showed that, in contrast to CYP1A1 and CYP2W1, CYP2S1 mediates the inactivation of these drugs [13,62]. A more detailed analysis of the metabolites formed from these drugs suggested that in its activation–deactivation, the hydroxylamine metabolite is involved, which can either be reduced back to the parent compound by CYP2S1 or progress to the formation of DNA adducts, mainly dGuo [62].

As was mentioned in the previous section, CYP2S1 expression was found in breast cancer cells differing in hormone receptor status. Interestingly, treating these cells with some methoxy-stilbenes or resveratrol increased the expression of CYP2S1 in ER(+) MCF7 cells, but not in ER(-) MDA-MB-231, where a decrease in the level of this favorable CYP was observed [14].

### 3.3. CYP2W1

Similarly to CYP2S1, the expression of CYP2W1 was described in breast cancer cells differing in hormone receptor status, and its upregulation was found in breast cancer samples [36]. Moreover, cells with higher levels of expression of these orphan CYPs were observed in MDA-MB-231 and MDA-MB-468 cells in comparison to ER(+) and PR(+) breast cancer cells.

The CYP2W1 gene is located on chromosome 7 p22.3 and encodes a protein comprising 490 amino acids [63]. The highest expression of CYP2W1 was noticed in the prostate and pancreas. CYP2W1 expression is, to a great extent, regulated through epigenetic mechanisms, mainly by DNA methylation. DNA hypermethylation is a pivotal epigenetic mechanism that silences many genes, including those regulating the cell cycle, inflammation, stress response, DNA repair, and apoptosis [64]. Hypermethylation of certain genes, particularly tumor suppressor genes, is known to be associated with the inactivation of various pathways involved in tumorigenesis. CYP2W1 gene expression is supposed to be regulated by epigenetic modification, namely DNA methylation of its promoter CpG islands. In this regard, the early study of Gomez et al. [65] showed that the expression of CYP2W1 in colon cancer was associated with the methylation status of its promoter and suggested a causal link between the gene CpG island’s demethylation and enhanced CYP2W1 expression. This suggestion is further supported by the fact that CYP2W1 is expressed in the course of development of the gastrointestinal tract, silenced after birth in the intestine and colon by its promoter CpG island’s hypermethylation, but activated following demethylation. Therefore, it seems that demethylation is a prerequisite for CYP2W1 expression, probably also in breast cancer cells [65].

CYP2W1 is induced by the AhR and ARNT pathway, as shown in experiments in which treatment of MDA-MB-468 and MCF7 breast cancer cells with AhR ligands such as 5F-203 and GW-610 increased expression of CYP2W1 [13,66]. Moreover, in contrast to CYP2S1, CYP2W1 was involved in the activation of these drugs [67].

Untargeted substrate searches showed that CYP2W1 catalyzes both hydroxylation and epoxidation of several fatty acids and phosphatidylcholine [68]. Moreover, among the numerous other endogenous substrates, there were also steroids, including 17β-estradiol. However, its binding to CYP2W1 was significantly weaker than that of retinoids [69]. CYP2W1 is involved in drug metabolism, facilitating such reactions as N-demethylation and aromatic hydroxylation, e.g., benzphetamine [70]. A high expression of CYP2W1 in tumor tissue, particularly in breast cancer (overexpression ~230-fold) in comparison to normal mammary gland tissues [33], makes this CYP isoform an attractive anticancer drug target. CYP2W1, having the ability to activate prodrugs, can increase the selectivity of chemotherapeutic agents to prevent tumor growth or metastasis as well as reduce the tumor volume before surgery [71]. Moreover, CYP2W1 can be a potential target for immunotherapy, since it is located on the cell membrane surface. A specific antibody, derived from the peptide sequence of CYP2W1, has already been developed and tested in MCF7 breast cancer cells [72].

### 3.4. CYP2U1

An immunohistochemical analysis, along with a survival analysis based on clinical–pathological features, showed that CYP2U1 is engaged in the malignant progression of breast carcinoma. Interestingly, the CYP2U1 protein level was inversely linked with the state of ER, i.e., much higher in ER(-) in comparison with ER(+) cancer tissue [37]. Therefore, it might be considered another druggable target for the treatment of advanced breast cancer. The human CYP2U1 gene is located on chromosome 4q25. Its protein product comprises 544 amino acids, with the region containing 8 proline residues before the transmembrane helix and an insert of about 20 amino acids rich in arginine residues located after the transmembrane helix [73]. CYP2U1 is the only member of this subfamily, and it seems to be very old and highly conserved across species [74]. Similarly to the orphan CYPs described above, CYP2U1 catalyzes fatty acid hydroxylation. These fatty acids include AA, docosahexaenoic acid, and eicosapentaenoic acids. CYP2U1-mediated metabolism of AA leads to the formation of 19- and 20-HETE. It was also shown that CYP2U1 efficiently catalyzes the hydroxylation of leukotriene B4 (LTB4) predominantly in its ω-position. The involvement of CYP2U1 in the metabolism of LTB4 could have significant physiological consequences, as LTB4 is an important inflammatory mediator involved in the pathogenesis of many diseases, including cancer [75]. Specific inhibitors of CYP ω-hydroxylases (e.g., 17-octadecenoic acid) decrease CYP2U1-mediated activity [37].

### 3.5. CYP4X1

As was mentioned in the previous section, CYP4X1, along with the orphan CYPs described above, showed the highest expression in breast cancer samples and correlation with the tumor grade [6]. The CYP4X1 gene is located in the cytochrome P450 ABXZ gene cluster along with CYP4Z2, and according to recent studies, this enzyme catalyzes epoxidation of endogenous cannabinoid anandamide and arachidonic acid [39,76]. Lower gene expression of CYP4X1 was associated with a shorter overall survival of Chinese gastric cancer patients treated with capecitabine and oxaliplatin [77].

A recent study by Hlavac et al. (2021) pointed out the association of a specific variant, rs17102977, in the CYP4X1 gene with the response of breast cancer patients to neoadjuvant cytotoxic chemotherapy. The substitution rs17102977 in the *CYP4X1* intron was associated with both the response of the patients to the neoadjuvant cytotoxic therapy and the disease-free survival of hormonally treated patients. It is also a prognostic in patients unselected according to the therapy. The endocannabinoid system is involved in various physiological processes, including inflammation, immunomodulation, and suppression of different cancers, including breast cancer; thus, CYP4X1 may play a role in the response to anticancer chemotherapy via physiological processes. The role of rs17102977 in cancer is, however, unknown [41].

## 4. Conclusions and Future Direction

Most of the orphan CYPs discussed in this review appear to be expressed in various cancer tissues, with only one, CYP4Z1, being specifically associated with the breast epithelium. Therefore, this CYP isoform may be considered the most promising orphan for breast cancer treatment. Current data suggest that inhibiting CYP4Z1′s breast-specific expression could slow down the growth, progression, angiogenesis, and invasiveness of breast cancer. Additionally, understanding the biological mechanism behind CYP4Z1 activation could help in the design of prodrugs that are selectively activated in breast tumor tissue, minimizing systemic side effects of chemotherapy. Moreover, the ability to reverse TAM resistance in CYP4Z1-positive breast cancer cells may serve as an important tool to improve existing adjuvant therapies. Finally, developing CYP4Z1 antibodies on the surface of breast cancer cells could aid in creating effective immunotherapies, such as anticancer vaccines. Since CYP4Z1 promotes breast cancer development by inducing ERα expression, its inhibition might have a dual benefit by removing a major risk factor for breast cancer.

**Figure 1:**
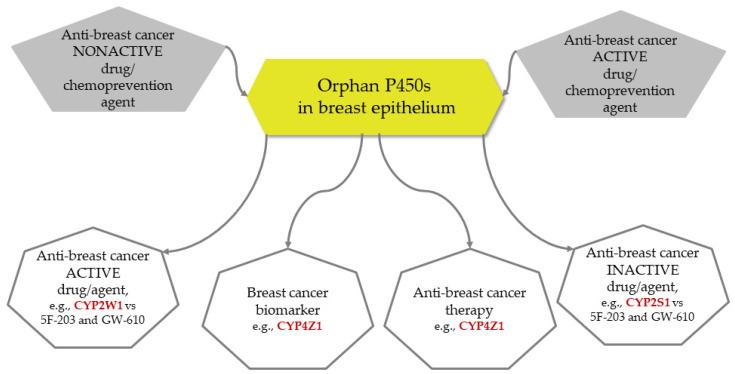
Proposed possible applications of orphan cytochromes P450 as targets in the treatment and prevention of breast cancer, 5F-203-2-(4-amino-3-methylphenyl)-5-fluorobenzothiazole, GW-610-2-(3,4-dimethoxyphenyl)-5-fluorobenzothiazole.

CYP2W1, CYP2S1, CYP2U1, and CYP4X1, although not as specific for the breast epithelium as CYP4Z1, show much higher expression in tumors than in normal tissues. Therefore, they may be considered more general cancer therapeutic targets or breast cancer progression biomarkers. The most promising are the results of the studies confirming the importance of these CYPs in TNBC cases, for which treatment options are limited, and the survival prognosis is poorer. Since their expression is not tissue-specific, the potential off-target effects when targeting these CYPs have to be taken into consideration. Possible directions in future studies and applications are suggested in Figure 1.

Although the functions and specific substrates of orphan CYPs remain uncertain, hence their orphan designation, it is clear that most of them catalyze fatty acid hydroxylation. Their products, such as EETs or HETEs, are considered critical modulators of cancer progression involved in promoting cellular proliferation, neovascularization, angiogenesis, and metastasis. Therefore, inhibition of the expression and activity of these orphan CYPs might be more useful in cancer treatment than in prophylaxis. On the other hand, CYP4Z1 induction of ERα expression may affect tumorigenesis initiation; thus, its inhibition may prevent tumor development. Therefore, further research aimed at the deorphanization of these cytochromes is justified and may credibly support the use of some orphan cytochromes in the prevention and therapy of breast cancer.

Unfortunately, knowledge about orphan CYPs and their role in breast cancer is limited to cell cultures and clinical studies involving a relatively small number of patients. Identifying endogenous ligands for these isoforms remains a current topic and appears to be crucial for understanding their role in breast epithelium homeostasis. Classical preclinical studies using mouse or rat models are limited for some of these isoforms because they are not expressed in these species. Therefore, there is a significant need to expand the range of cell lines and conduct clinical trials with large, well-characterized patient groups. Additionally, patient-derived models—such as patient-derived cell cultures, spheroids, organoids, tissue slice cultures, or xenografts—offer valuable opportunities [78]. Studies that assess both the transcript and protein levels of orphan CYPs, along with the presence of antibodies on the surface of normal and cancerous breast epithelial cells, could provide important insights.

In conclusion, orphan CYPs expressed in both tumor and nontumor breast tissues may be considered potential targets for inhibiting tumorigenesis, particularly the delay of breast cancer progression. The orphan CYP isoforms influencing the activation of potential prodrugs and sensitizing cells resistant to adjuvant therapy bring hope for the effective prevention and treatment of breast cancer. Additionally, the overexpression of some orphan CYPs in breast cancer cells related to ER status may be useful for monitoring cancer progression. Further, more-in-depth studies are needed to confirm these ideas, with a particular emphasis on the indication of the substrate and metabolic specificity of the CYP450 enzymes in question.

## Figures and Tables

**Table 1:** In vitro studies on the orphan CYPs in human breast epithelial cell lines.

Orphan CYP450	In Vitro Model (Cell Line)	Treatment	Findings	References
2S1	MCF10A	n/t	mRNA detected predominantly or exclusively in sub-confluent cultures	[23]
	MCF7 MDA-MB-468	n/t n/t n/t Exogenous AhR ligands	mRNA in both cell lines higher expression in MDA-MB-468 low protein level induced expression	[13]
	MCF7 MDA-MB-231	n/t n/t Synthetic methoxystilbenes Resveratrol and synthetic methoxystilbenes (3MS, 4MS, 5MS)	mRNA and protein in both cell lines higher expression in MDA-MB-231 increased expression in MCF7 decreased mRNA in MDA-MB-231 cells	[14]
2U1	MCF10A	n/t	mRNA detected in both sub-confluent and confluent cultures	[23]
2W1	MCF7 MDA-MB-468	n/t n/t Exogenous AhR ligands	mRNA in both cell lines higher expression in MDA-MB-468 induced expression	[13]
	MCF7 MDA-MB-231	n/t n/t Synthetic methoxystilbene 3MS Resveratrol and synthetic methoxystilbenes (3MS, 4MS, 5MS)	mRNA and protein in both cell lines higher expression in MDA-MB-231 increased protein in MCF7 decreased mRNA in MDA-MB-231 cells	[14]
4V2	MCF10A	n/t	mRNA detected in both sub-confluent and confluent cultures	[23]
4X1	MCF10A	n/t	mRNA detected in both sub-confluent and confluent cultures	[23]
4Z1	MCF7	n/t	mRNA and protein in breast tissue (normal and cancer) with low expression levels (in comparison to other human CYPs) in other tissues (e.g., liver)	[15]
	MCF7 T47D	n/t	mRNA preferentially expressed in mammary tissue implication of progesterone and glucocorticoid receptor in CYP4Z1 gene activation	[16]
	MCF10A	n/t	mRNA was detected in both sub-confluent and confluent cultures	[23]
	T47D BT-474	n/t	immunostaining overexpression promotes tumor angiogenesis and growth in breast cancer	[21]
	MCF7 and MCF7-TamR	n/t n/t n/t	CYP4Z1 and CYP4Z2P downregulated in MCF7 compared with MCF7-TamR overexpression of CYP4Z1- or CYP4Z2P-3′ UTR enhances transcriptional activity of ERα blocking of CYP4Z1- and CYP4Z2P-3′ UTR reversed tamoxifen resistance in MCF7-TamR	[25]
	MCF7 MDA-MB-231	n/t	downregulation of CYP4Z1- or CYP4Z2P-3′ UTR promotes cell apoptosis	[26]
	MCF7 MDA-MB-231	n/t	comprehensive endogenous RNA network mediated by CYP4Z1 gene and CYP4Z2P pseudogene promoted stemness of breast cancer	[17]
	MCF7 MDA-MB-231	n/t	overexpression of CYP4Z1 3′ UTR could suppress capacity of migration and adhesion of these cells by acting as competitive endogenous RNAs for E-cadherin	[18]
	MCF7	n/t n/t	demonstrate presence of CYP4Z1 enzyme on outer surface of plasma membrane of MCF7 detection of high titers of anti-CYP4Z1 autoantibodies in breast cancer patients but not in healthy controls	[19]
	MCF10A	n/t	no display of CYP4Z1 on MCF10A cells’ surface	[24]
	T47D transfected with CYP4Z1	Novel synthetic ‘7’ inhibitor	inhibition of 14,15-EET (product of arachidonic acid metabolism, influencing proliferation, migration, and angiogenesis)	[22]
	combination of in vitro and silico models of recombinant CYP4Z1 mutants	n/t	Arg487 and Asn381 residues in CYP4Z1 protein play crucial role in substrate recognition and binding	[28]
	MCF7 MDA-MB-231	HET0016	synthetic CYP4Z1 inhibitor CYP4Z1 promoted stemness of MCF7 breast cancer cells	[29]
	MCF7	Novel CYP4Z1 inhibitors	discovery of novel CYP4Z1 inhibitors in enzyme bag test and CYP4Z1-overexpressing MCF7 cell clone	[30]
	MCF7 BT549 SUM159 MDA-MB-231	n/t 20-HETE	CYP4Z1 mRNA expression 20-HETE treatment promoted growth of TNBC cell lines (BT549, SUM159, MDA-MB-231)	[20]

3’ UTR—3’ untranlated region, 3MS—methoxy-stilbenes, 3,4,2′-trimethoxy-*trans*-stilbene; 4MS—3,4,2′,4′-tetramethoxy-*trans*-stilbene; 5MS—3,4,2′,4′,6′-pentamethoxy-*trans*-stilbene; 20-HETE—20-hydroxyeicosatetraenoic acid; N-hydroxy-N’-(-butyl-2-methylphenyl)-formamidine; n/t—not-treated, TamR—tamoxifer resintant.

**Table 2:** Clinical studies on the orphan CYPs in breast cancer patients.

Orphan CYP450	Patients Pool	Results/Conclusions	References
2A7	20 tumor and control breast tissue samples	no mRNA detected	[34]
	165 triple-negative breast cancer samples	expression associated with poorer survival	[35]
2S1	170 breast cancer, no special-type samples	37.5% of CYP2S1 immune-positive cells absence of CYP2S1 correlated with better survival	[6]
	50 breast cancer patients and 31 controls	mRNA increased protein not detected	[32]
	1426 early-stage invasive breast cancer	low immunohistochemical protein expression associated with poorer patient survival	[36]
2U1	170 breast cancer, no special-type samples	32.2% of CYP2S1 immune-positive cells correlated with tumor grade	[6]
	219 invasive breast cancer	high immunohistochemical protein level correlated with poorer survival more frequent in TNBC	[37]
2W1	32 breast cancer patients and 20 controls	mRNA expressed in breast cancer, adjacent, and normal breast cells 230 times higher in breast cancer than in normal breast cells expression associated with Ki67	[33]
	50 breast cancer patients and 31 controls	significantly overexpressed in tumors higher 2W1 mRNA correlated with better response to neoadjuvant chemotherapy not confirmed on protein level (too low)	[32]
	1426 early-stage invasive breast cancer	low immunohistochemical protein expression associated with poorer patient survival	[36]
3A43	170 breast cancer, no special-type samples	70.7% of samples most frequently displayed no immunoreactivity	[6]
	1143 incident breast cancer cases and 1155 population controls	allele CYP3A43_74_delA correlated with higher-grade breast tumors	[38]
4F11	32 breast cancer patients and 20 controls	mRNA expressed in breast cancer, adjacent, and normal breast cells no statistical differences between cancer and normal tissue expression associated with Ki67	[33]
4V2	170 breast cancer, no special-type samples	immunostaining was correlated with survival correlated with tumor grade	[6]
4X1	one individual patient	mRNA detected	[39]
	170 breast cancer no special-type samples	50.8% of CYP4X1 immune-positive cells immunostaining correlated with lower tumor grade	[6]
	120 primary breast cancer and 5 nontumorigenic controls	off-frame fusion with pseudogene CYP4Z2P of unknown function	[40]
	105 breast cancer patients with neoadjuvant cytotoxic chemotherapy	variant rs17102977 in CYP4X1 associated with response to neoadjuvant cytotoxic chemotherapy	[41]
4Z1	54 breast tumors	mRNA overexpression (microarray) in 50% of samples	[42]
	170 breast cancer, no special type samples	immunostaining correlated with increasing tumor grade	[6]
	249 breast cancer patients ER(+)	immunostaining correlation between mutated oncogene PIK3CA and overexpression of CYP4Z1 and pseudogene CYP4Z2P	[43]
	paraffin-embedded breast cancer tissue samples and 8 pairs of fresh breast cancer and normal tissues	comprehensive endogenous RNA network mediated by CYP4Z1 gene and CYP4Z2P pseudogene promoted stemness of breast cancer	[17]
	sera from 19 breast cancer patients and 11 control sera	demonstrate presence of CYP4Z1 enzyme on plasma membrane of MCF7 detection of high titers of anti-CYP4Z1 aAbs in breast cancer patients but not in healthy controls	[19]
	220 breast cancer cases and 8 normal breast tissues	immunohistochemically, 82% of malignant samples with moderate–intense expression normal tissues and benign tumors: no-to-weak expression	[44]
	122 TNBC cases and 4 normal breast tissues	strong expression of CYP4Z1 (83.3%) in various TNBC subtypes negative expression in normal samples poorer overall survival of TNBC patients with high CYP4Z1 expression in comparison to patients with low CYP4Z1 expression	[31]
	5 TNBC patients	patient-derived xenografts expressed CYP4Z1 mRNA	[20]
	86 anthracycline-responsive breast cancer patients vs. 7 anthracycline-non-responsive	CYP4Z1 was significantly upregulated in anthracycline-resistant group	[45]

## Data Availability

No new data were created or analyzed in this study.

## Abbreviations

The following abbreviations are used in this manuscript:

AA	arachidonic acid
AhR	aryl hydrocarbon receptor
ARNT	AhR nuclear translocator
CYPs	cytochromes P450
EET	epoxyeicosatrienoic acid
ER	estrogen receptor
HET0016	N-hydroxy-N’-(-butyl-2-methylphenyl)-formamidine
HETE	hydroxyeicosatetraenoic acid
HER	human epidermal growth factor receptor 2
LTB4	leukotriene B4
PR	progesterone receptor
PUFAs	polyunsaturated fatty acids
TAM	tamoxifen
TNBC	triple-negative breast cancer
